# Serratus anterior muscle flap as a salvage procedure in exposed implant-based breast reconstruction

**DOI:** 10.1016/j.ijscr.2019.08.033

**Published:** 2019-09-13

**Authors:** Eduardo Montag, Alberto Okada, Eduardo G.P. Arruda, Alexandre M. Munhoz, Fabio F. Busnardo, Rolf Gemperli

**Affiliations:** a Division of Plastic Surgery, University of São Paulo School of Medicine, Brazil; b São Paulo Cancer Institute – University of São Paulo, Brazil

**Keywords:** Mammaplasty, Surgical flaps, Breast neoplasms, Breast implants

## Abstract

**Background:**

Implant-based breast reconstruction (IBR) is the most common approach to reconstruct mastectomy deffects. Infection following breast reconstruction can be devastating and lead to loss of the reconstruction due to the need of implant removal. The serratus anterior muscle flap is widely used during breast reconstruction to provide coverage of the implant/expander.

**Methods:**

We present the application of the serratus anterior muscle flap to treat implant exposure after breast reconstruction.

**Cases presentation:**

Two patients who experienced implant exposure after breast reconstruction were successfully treated with partial capsulectomy, pocket irrigation and implant coverage with a serratus anterior muscle flap.

**Results:**

No post operative complications have been observed while implant retention was achieved in both cases.

**Conclusions:**

The serratus anterior muscle flap is an option to treat implant exposure following breast reconstruction in selected cases. This flap could be used to prevent implant exposure in critical cases.

## Highlights

• Implant based breast reconstruction is the most popular form of breast reconstruction following cancer treatment.
• Implant exposure normally leads to the loss of reconstruction due to contamination and infection.
• The two case series illustrate an approach to implant exposure with a novel use of the serratus anterior muscle flap. We discuss the main indications and technical aspects which can be helpful for surgeons dealing with similar cases.

## 1 Introduction

Implant based reconstruction is the most common approach for patients seeking reconstruction following mastectomy [[1], [2], [3]]. The devices are readily available and provide reconstruction with high satisfaction levels. Radiotherapy is also being used increasingly leading to increased rates of capsular contracture and implant loss [4]. Traditionally expander to implant exchange is performed months after the end of radiotherapy. However, this kind of approach is not free from adverse events such as skin necrosis, implant exposure and infection. Implant exposure is a situation that leads to a surgical emergency due to the risk of implant loss [2,5,6]. Patients experiencing implant exposure normally require implant removal followed by delayed reconstruction which can be challenging due to the presence of radiation therapy effects [7]. Although it is a fairly common situation there is little evidence that salvage procedures are safe and should be performed [5,7]. Latissimus dorsi flap can be used in the setting of implant exposure but in case of failure one loses the opportunity for a delayed reconstruction using this method. Up to now the serratus anterior muscle has been widely used to cover the inferior lateral portion of tissue expander and implants during immediate reconstruction [8]. We extended the use in the setting of wound dehiscence to treat implant exposure.

## 2 Clinical cases presentation

From April to June 2015, 2 patients presenting with wound dehiscence and actual or threatened implant exposure without evidence of severe infection after expander or implant exchange have been treated by means of a serratus anterior muscle flap without implant removal.

## 3 Methods

The study was approved by the review board at the and conducted in accordance with the Declaration of Helsinki. It can be found on researchregistry.com under the number 4881. The research work has been reported in line with the PROCESS criteria [9].

This is a retrospective, single centre, non consecutive case series. The patients where treated in a public, academic, tertiary, hospital.

### 3.1 Case 1

A 51-year old patient with a history of bilateral mastectomy and expander based reconstruction followed by chemotherapy and radiotherapy. After two years she underwent bilateral implant exchange. Approximately 30 days later she presented with implant exposure without drainage. She was submited to debridment, parcial capsulectomy, implant exchange and serratus anterior muscle flap for coverage. Cultures showed *s. epidermidis.* The post operative course was uneventful and was discharged at post op day 5 with oral antibiotics. The patient remained infection free through a 3 year follow up.

### 3.2 Case 2

**Fig. 1:**
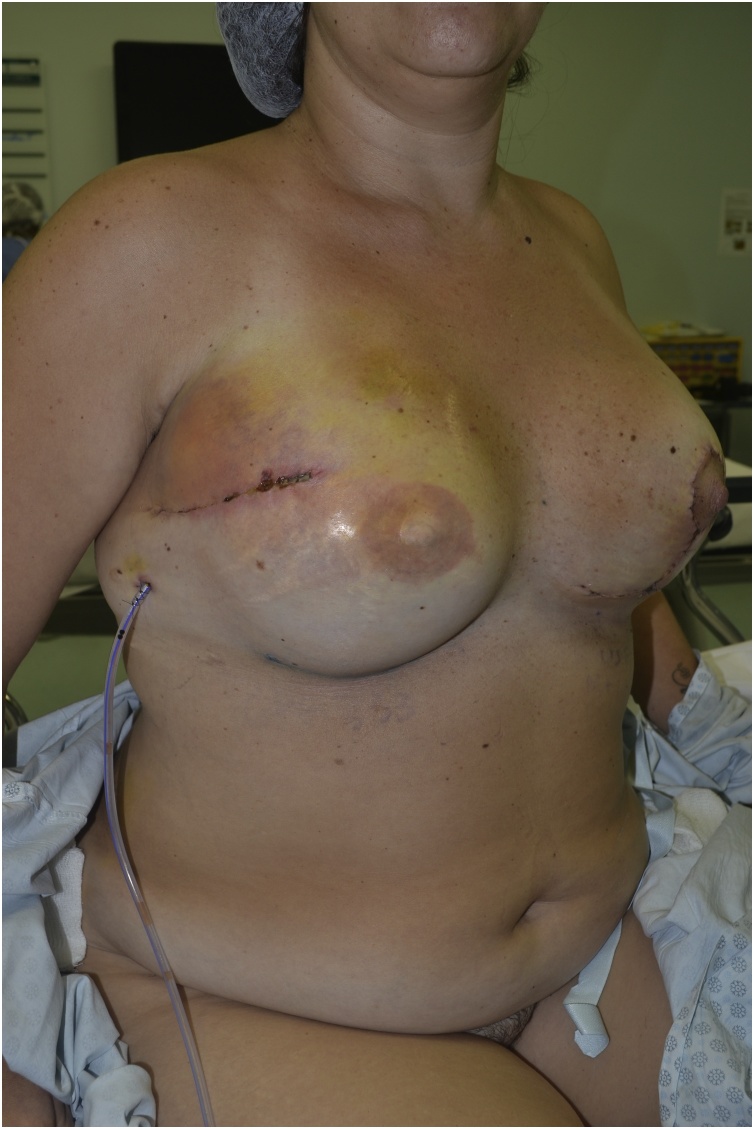
6 month follow up (frontal view).

**Fig. 2:**
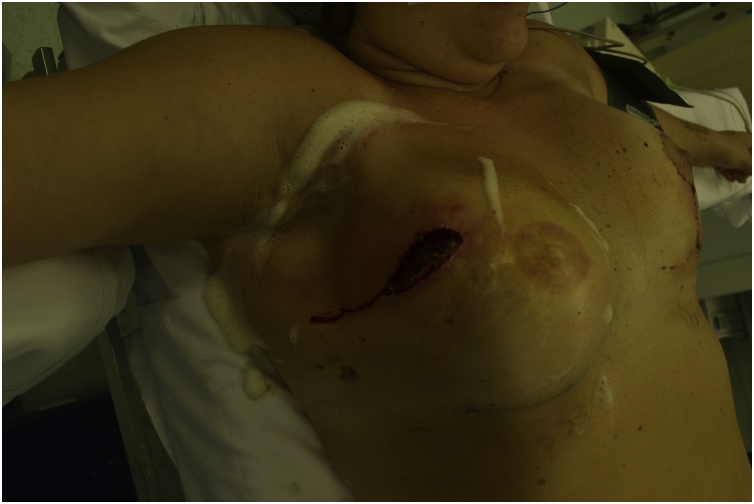
Implant exposure.

**Fig. 3:**
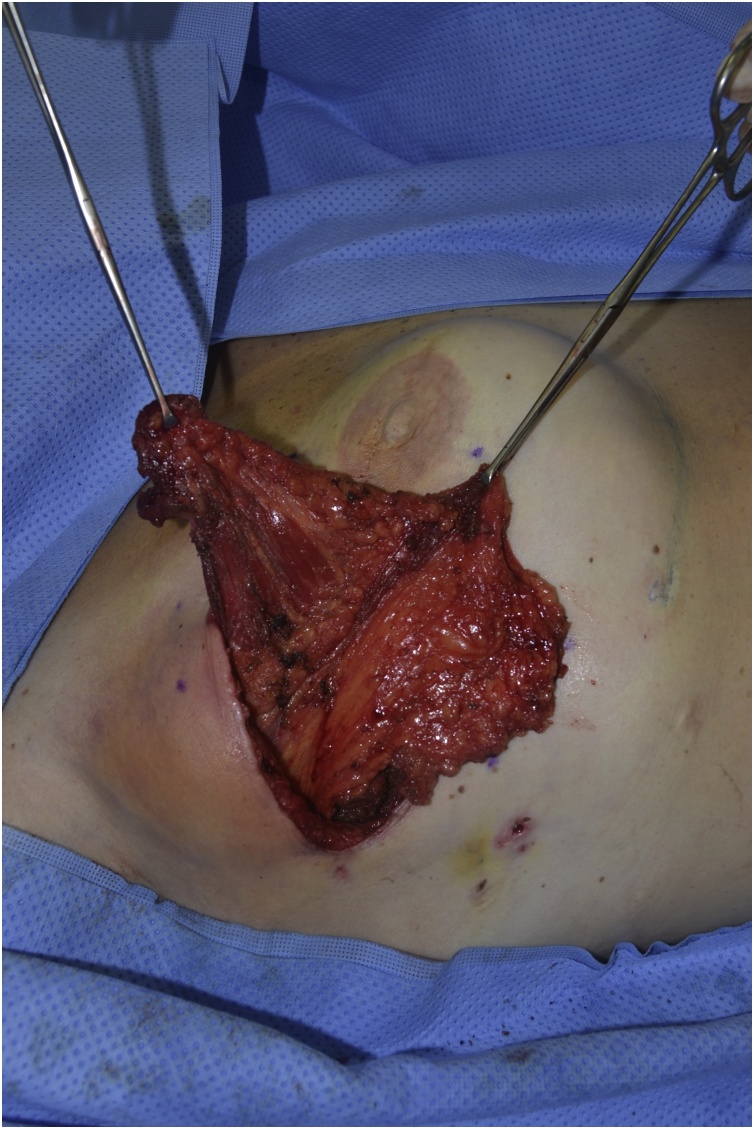
Serratus muscle exposed through surgical incision.

**Fig. 4:**
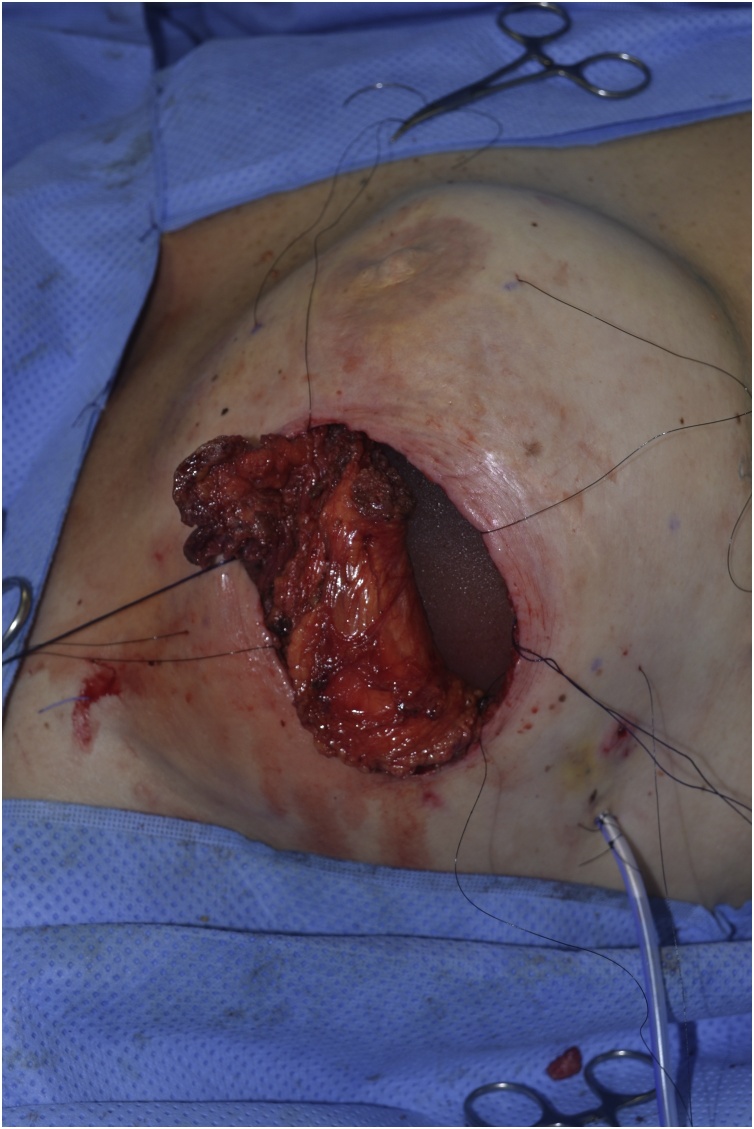
Implant partially covered by flap.

**Fig. 5:**
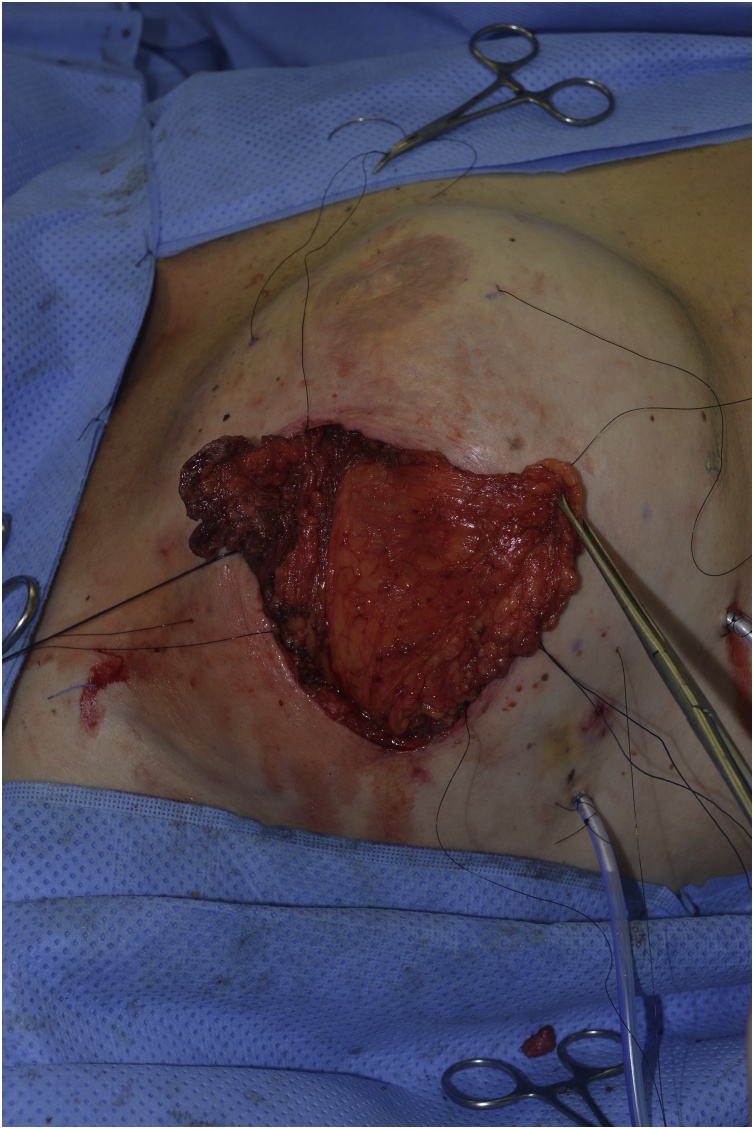
Total muscle coverage.

**Fig. 6:**
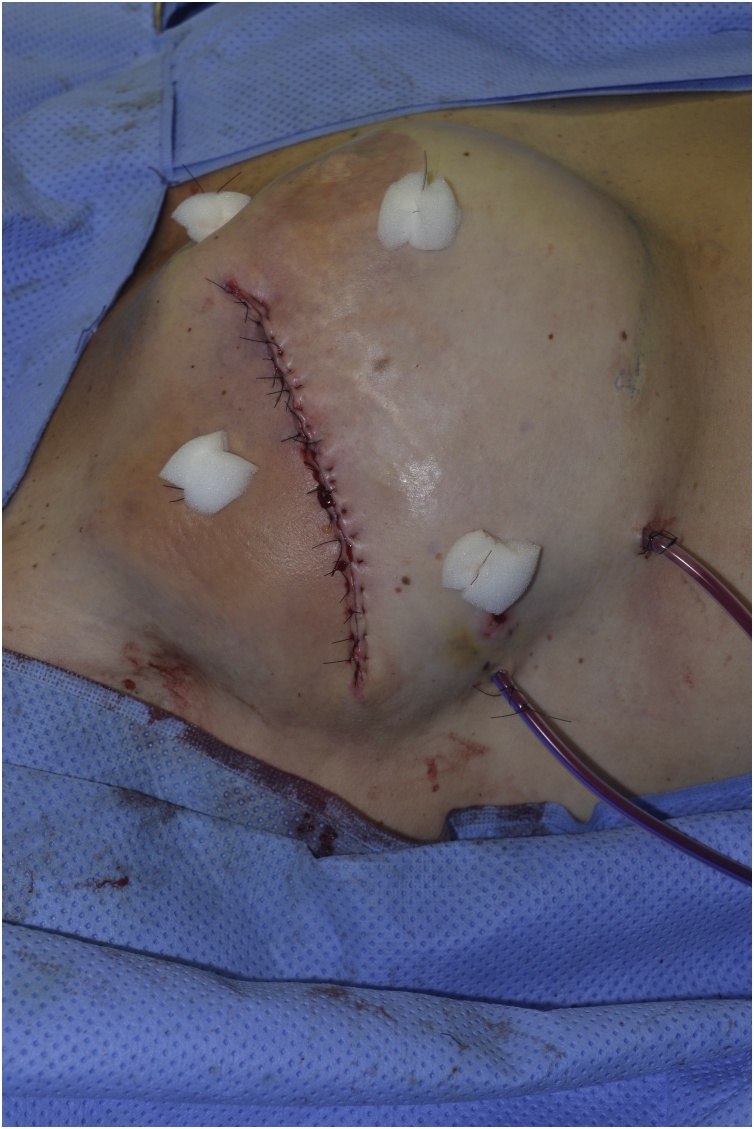
Imediate post operative.

**Fig. 7:**
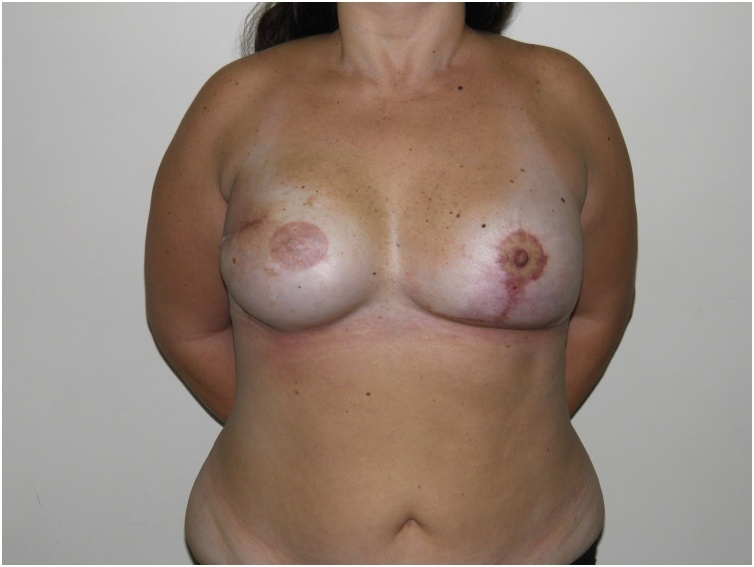
Patient at fifth post operative day after implant exchange and lipofilling. Implant exposure.

**Fig. 8:**
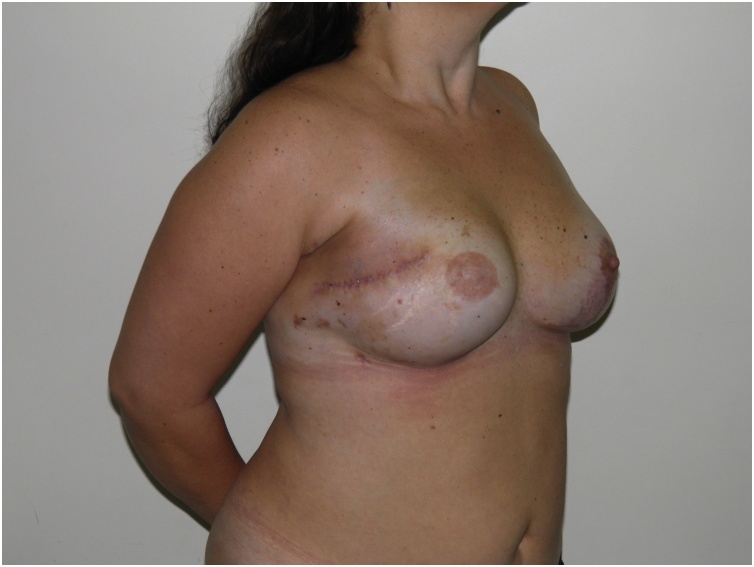
6 month follow up (oblique view).

A 38-year old patient with a history of right mastectomy, expander reconstruction, radiation therapy and recurrent capsular contracture. One week after capsulotomy, implant exchange and fat grafting she presented with erythema of the lateral portion of the breast and thinning of the surgical scar which was interpreted as a threatened exposure with mild infection. Operative steps included pocket irrigation with antibiotic saline solution, pocket enlargement, placement of two suction drains and anterior reinforcement with a serratus anterior muscle flap. Erythema and swelling showed improvement and the patient was discharged on post operative day 4. Cultures showed no sign of bacterial growth. She was given oral antibiotics for 2 weeks and remained infection free through 14 month follow up Fig. 1, Fig. 2, Fig. 3, Fig. 4, Fig. 5, Fig. 6, Fig. 7, Fig. 8,).

## 4 Surgical procedure

The procedure is done under general anesthesia by the senior author with assistance from the residents. According to hospital protocol the patients receive wide spectrum antibiotics before surgery starts. With the patient in supine position with arms abducted at 90°, disinfection of the area is performed. Skin margins are removed until adequate bleeding is observed. After implant removal, swabs and tissue samples are obtained for microbiological culture. The implant pocket is thoroughly irrigated with saline and antibiotic solution to reduce the chance of biofilm formation. A lateral capsulotomy is performed and dissection proceeds posteriorly until the anterior edge of the serratus muscle is exposed. From there the muscle dissected from the rib cage up to the posterior axillary line. With the aid of three forceps the muscle is pulled anteriorly and released from the superficial tissue. Care must be taken in order to avoid any damages to the vascular pedicles of both the serratus and the latissimus dorsi muscles. After enough bulk is obtained to cover the implant and reinforce the skin suture the muscle is the split and rotated anteriorly to cover the implant. Whenever possible the implant is replaced by a smaller one to facilitate skin closure. After adequate hemostais is obtained two suction drains are placed, the muscle is sutured to the anterior capsule and the skin is closed. The average operating time was 55 min (range). Drains were removed after 5 to 7 days.

## 5 Discussion

The management of an exposed breast implant normally requires the device removal, antibiotic therapy and delayed reconstruction [5,7]. Salvage of an exposed and/ or infected breast implant has always been a challenge for most of the patients, especially the ones with previous radiation therapy.The concept was first introduced by Perras in 1965 [6]. After that many authors contributed to a change in the idea that this kind of device has always to be removed to ensure adequate control of the infectious process. Courtiss at al. were the first to publish on the subject specifically for reconstruction patients [6]. Adequate systemic antibiotic therapy has an important role in controlling the infection once the diagnosis is made and culture samples are obtained to guide it. Implant pocket treatment with antibiotic solution, capsulotomies, curettage and implant exchange were also added to improve the chances of success. Spear, in 2004, described an algorithm for implant infection management providing another valuable tool for plastic surgeons dealing with this potentially devastating situation [5]. In 2009 Spear further enhanced the knowledge of when and how to attempt reconstruction salvage and introduced the concept of flap coverage to enhance soft tissue coverage and reduce the chance of a new exposure [6]. Recently the use of negative pressure and antibiotic impregnated plates has been described with success to improve fluid drainage and local delivery for drugs and can be promising factors for reconstruction salvage in the future [16,17].

Some authors advocate for site change or flap reinforcement to minimize the odds of a new skin breakdown [10]. The most common muscle used for this purpose in breast reconstruction is the latissimus dorsi flap [[11], [12], [13]]. Although it can be performed through the site of implant exposure it normally requires a dorsal scar, additional morbidity and, in case of infection relapse the best option for a delayed reconstruction is lost [14]. It is our understanding that no bridges should be burned during salvage attempts therefore the latissimus flap should be spared to be used for delayed reconstruction of failure to save the exposed implant.

Rempel reported the use of a small serratus anterior muscle flap as a way to cover an exposed breast implant after aesthetic augmentation with no success [15]. Later he was cited by Wilkinson that described a two flap technique for implant coverage containing parts of the pectoralis major and serratus anterior muscles combined with a limberg skin flap [6]. Other authors report the use of the serratus fascia as an alternative to the muscle flap in order to reduce donor site morbidity [17,18]. This approach can be used for immediate reconstruction patients but is limited in patients submitted to reoperations or previously irradiated.

We report the use of a large muscle pad as a way to reinforce the skin suture, reduce the chance of wound breakdown and a new exposure which would lead to a failure of the reconstruction. The drawback with this flap is the lack of a cutaneous portion to be used in cases where more room is needed to accommodate a large implant. It should be regarded as a valid option also for prevention of implant exposure in patients with extremely thin and irradiated flaps [19] None of the patients presented complications from the harvesting of the muscle flap other than pain located at the donor site. One of the patients has breast asymmetry due to implant volume difference and is waiting for lipofilling to correct the deformity.

## 6 Conclusions

Salvage of the exposed breast implant remains a challenging situation for surgeons and patients. Keys to success include agressive management of the implant pocket, antibiotics, device exchange, and adequate soft-tissue coverage. The serratus anterior muscle flap represents a simple, versatile and low morbidity option for this group of patients.

This flap should also be regarded as a valid surgical option for patients prone to implant exposure and failure of the reconstruction such as those presenting with extremely thin skin flaps or radiation damage prior to implant exchange.

## Sources of funding

We have no sources of funding and sponsor for the present research.

## Ethical approval

The present case report was approved by Plastic Surgery Department of the Hospital das Clínicas of the University of São Paulo.

## Consent

Written informed consent was obtained from the patients for publication of this paper and accompanying images.

## Author contributions

Eduardo Montag: study concept or design, data collection, data analysis or interpretation, writing the paper.

Alberto Okada: data collection, data analysis.

Eduardo Gustavo Arruda: data analysis.

Alexandre Mendonça Munhoz: interpretation. Fábio F. Busnardo: manuscript revision

Rolf Gemperli: data analysis, interpretation.

## Registration of research studies

The study was registered at research registry under the number research registry 4881 as stated in the paper.

## Guarantor

Eduardo Montag.

## Provenance and peer review

Not commissioned, externally peer-reviewed.

We have no conflict of interest, financial and personal relationships with other people or organisations that could inappropriately influence (bias) our present work.

## Declaration of Competing Interest

We have no conflict of interest, financial and personal relationships with other people or organisations that could inappropriately influence (bias) our present work.
